# Targeting GSTZ1 Sensitizes *KRAS^G12C^*-Mutant Lung Cancer Cells by Overcoming Glutathione and Glycolysis Pathway Rewiring

**DOI:** 10.1158/2767-9764.CRC-25-0698

**Published:** 2026-06-11

**Authors:** Yi Liao, Xueli Li, Min Liu, Vanessa C. Fernandes, Derek Duckett, Eric B. Haura, Andrii Monastyrskyi, Uwe Rix

**Affiliations:** 1 Department of Drug Discovery, H. Lee Moffitt Cancer Center & Research Institute, Tampa, Florida.; 2 Proteomics and Metabolomics Core, H. Lee Moffitt Cancer Center & Research Institute, Tampa, Florida.; 3 Department of Thoracic Oncology, H. Lee Moffitt Cancer Center & Research Institute, Tampa, Florida.; 4 Department of Molecular Biosciences, University of South Florida, Tampa, Florida.

## Abstract

*KRAS* mutations are prevalent in lung cancer, but KRAS^G12C^ inhibitors exhibit limited efficacy, partly due to metabolic adaptations, such as enhanced glutathione (GSH) metabolism and increased glycolysis. Glutathione S-transferase zeta 1 (GSTZ1) is a metabolic enzyme that regulates cell metabolism; however, its role in *KRAS*-driven lung cancer remains underexplored. We recently reported that targeting *GSTZ1* significantly enhances the efficacy of FDA-approved KRAS^G12C^ inhibitors in non–small cell lung cancer (NSCLC) cells. Untargeted metabolomics now revealed significant alterations in GSH and glycolytic pathways, marked by lowered lactate levels and increased oxidized GSH following *GSTZ1* ablation. Moreover, pharmacologic inhibition of GSH synthesis and glucose uptake mimicked the sensitizing effects of *GSTZ1* targeting. These metabolic shifts were accompanied by increased AMPK phosphorylation and reduced AKT phosphorylation, two key mediators of the response to KRAS^G12C^ inhibition. Our data reveal GSTZ1-associated metabolic and signaling alterations that contribute to drug resistance and identify GSTZ1 as a potential complementary target to sensitize *KRAS*-mutant NSCLC to KRAS-directed treatments.

**Significance:**

Targeting GSTZ1 sensitized *KRAS*^*G12C*^-mutant NSCLC cells to KRAS^G12C^ inhibitors by disrupting glycolysis, GSH metabolism, and protein phosphorylation. GSTZ1 emerges as a mediator of drug resistance and a therapeutic target, supporting rational combination strategies that exploit metabolic vulnerabilities to enhance KRAS-targeted therapy efficacy and improve outcomes.

## Introduction

Targeted therapies have significantly benefited patients with non–small cell lung cancer (NSCLC), including those with tumors harboring a *KRAS*^*G12C*^ mutation. Despite the clinical availability of KRAS^G12C^-targeting inhibitors, such as sotorasib and adagrasib, only approximately 30% to 50% of patients show a favorable response due to intrinsic resistance mechanisms (1). Drug refractoriness commonly develops during treatment, which limits the upfront and long-term effectiveness of these KRAS^G12C^ inhibitors (2, 3). Preclinical studies and clinical observations have revealed a range of resistance mechanisms, including heterogeneous *KRAS* mutations, variable *KRAS* expression, and persistent RAS signaling dependencies (2–4).

In addition to alterations in RAS family proteins and signaling rewiring, metabolic reprogramming is a critical contributor to drug response, particularly metabolic pathways that maintain redox homeostasis and support glycolysis under therapeutic stress (5). Cancer cells, including *KRAS*-mutant NSCLC, increase their reliance on antioxidant systems and glutathione (GSH) metabolism to counteract elevated reactive oxygen species (ROS) in tumorigenesis (6–8). Oncogenic *KRAS* mutations can promote glycolysis, thereby increasing glucose uptake that fuels biosynthetic pathways crucial for tumor growth (9). This metabolic shift enables cancer cells to mitigate the effects of KRAS inhibitors by sustaining energy production and facilitating alternative survival mechanisms (10). Moreover, metabolic plasticity can render tumors resistant even in the process of drug treatment and pose a significant challenge to achieving durable treatment responses to KRAS-targeted therapies.

The glutathione S-transferase (GST) protein family in humans acts as vital antioxidant enzymes and regulators of stress-induced signaling. Overactive GST proteins are often found in various cancers and are involved in key tumorigenic processes such as cell survival, proliferation, and drug resistance (11). Many GST enzymes focus on detoxifying harmful compounds through conjugation with GSH; however, GSTZ1 primarily functions as a maleylacetoacetate isomerase that uses GSH as a catalytic cofactor, catalyzing the isomerization of maleylacetoacetate to fumarylacetoacetate in the terminal steps of tyrosine and phenylalanine catabolism (12). This specialized activity allows GSTZ1 to directly contribute to amino acid catabolism and modulate cellular redox buffering capacity. Mechanistically, perturbation of GSTZ1 activity can alter levels of reactive intermediates and GSH availability, with downstream consequences for oxidative stress signaling and metabolic flux (13). For instance, GSTZ1 was recently reported to regulate redox balance and inflammation via modulation of the NRF2/GPX4 axis in hepatocellular carcinoma (14) and the HMGB1/GPX4 axis to drive ferroptosis in bladder cancer cells (6). Beyond its classic functions, our previous chemoproteomics studies revealed that GSTZ1 constitutes a druggable vulnerability in NSCLC and is involved in the cell survival and drug response signaling, such as FGFR1, particularly in the context of *KRAS*^*G12C*^ mutation (15). Taken together, these biochemical and biological links between oncogenic and redox signaling pathways strongly support the rationale for investigating GSTZ1 as a modulator of therapeutic response in *KRAS*-mutant tumors.

AMP-activated protein kinase (AMPK) is a critical regulator of cancer cell metabolism and stress responses by reprogramming cellular energy homeostasis toward suppressing tumor growth (16). AMPK activation inhibits key anabolic processes, for instance, through downregulation of the mTOR pathway, while simultaneously inducing energetic stress and triggering metabolic reprogramming and apoptosis. These effects provide a robust framework for developing AMPK-activating targeted therapies against cancer (17, 18). Glycolysis inhibition and oxidative stress are critical in activating AMPK in lung cancer cells. Glycolysis inhibition can reduce ATP production and elevate the AMP/ATP ratio, thereby activating AMPK to initiate catabolic pathways and inhibiting anabolic processes to conserve energy (19). Oxidative stress is reported to enhance AMPK activation through phosphorylation at Thr172 which is crucial in lung cancer metabolic adaptation (20, 21). Therefore, glycolytic inhibition and oxidative stress–induced activation of AMPK signaling provides therapeutic leverage points for developing targeted therapies. There are preclinical and clinical studies highlighting the potential of AMPK activators to enhance the efficacy of conventional chemotherapeutics, overcome drug resistance, and improve patient outcomes in cancer therapy (22–26); for example, a randomized phase II study evaluated an AMPK activator metformin, which is approved for type 2 diabetes, combined with paclitaxel/carboplatin/bevacizumab in NSCLC, supporting further study of metformin as a metabolic adjuvant to enhance anticancer drug efficacy (23). However, there are currently no AMPK activators approved for cancer therapy, alone or in combination with KRAS inhibitors, and further research into alternative approaches to target this pathway is warranted. In this study, we report that *GSTZ1* targeting disrupts glycolysis and redox homeostasis and AMPK signaling in *KRAS*^*G12C*^-mutant NSCLC cells, highlighting promising avenues for combined therapeutics that target both GSTZ1 and KRAS.

## Materials and Methods

For details on cell lines and reagents, also see the Key Resource Table (Supplementary Table S1).

### Cell culture and maintenance

HBEC-30KT (27), H1792 (28), H358 (28), LU99 (28), H2122 (28), HOP62 (28), Calu-1 (28), HCC1171 (29), and MRC-5 (30) were obtained from the Moffitt Lung Cancer Center of Excellence cell line core. All cell lines have been authenticated by short tandem repeat analysis and tested negative for *Mycoplasma* using the PlasmoTest Mycoplasma Detection Kit (InvivoGen). HBEC-30KT was cultured in the DMEM media, and others were cultured in RPMI 1640 media supplemented with 10% fetal bovine serum and maintained in a humidified atmosphere containing 5% CO_2_ at 37°C. Cells were cultured and used within 3 months after being revived from frozen aliquots.

### Cell viability measurements

Cells were seeded in 96-well black-walled/clear-bottom microtiter plates (Corning) at a density of 2,000 to 3,000 cells per well and treated with drugs and/or *GSTZ1*-targeted siRNAs for indicated period of time following 24-hour cell attachment. Cell viability was measured using CellTiter-Glo (Promega) and based on the manufacturer’s protocol. Plates were read on the M5 SpectraMax plate reader (Molecular Devices) with 500 milliseconds integration. For cell viability measurements in six-well plates, cells were trypsinized, harvested, washed, and resuspended in 1 mL of RPMI 1640 culture media. Cell suspension (20 μL) was transferred in triplicate to microcentrifuge tubes, followed by trypan blue staining and counting of viable cells. Data were processed and analyzed using Excel and GraphPad Prism 10.0.

### RNA interference

SMART pool *GSTZ1* (Horizon, L-011290-00-0005) and ON TARGET plus nontargeting (Horizon, D-001810-10-20) siRNAs were used. siRNAs were resuspended in 1× siRNA buffer diluted with RNase-free water, aliquoted, and stored at −80°C. siRNA stocks were thawed on ice, diluted with Opti-MEM (Thermo Fisher Scientific, 31985062), and mixed well with Lipofectamine RNAiMAX (Thermo Fisher Scientific, 13778150). Transfection of siRNAs (25 nmol/L) was performed in either six-well plates or 96-well plates. Cells were plated with a density of 3 to 5 × 10^5^ cells per well for six-well plates and 2,000 to 3,000 cells per well for 96-well plates and treated with siRNAs or drugs for the indicated time.

### CRISPR-Cas9–mediated *GSTZ1* knockout

Genetic experiments in cell lines were approved by the Institutional Biosafety Committee at the University of South Florida. Two *GSTZ1*-targeted single-guide RNA (sgRNA), sgRNA1: 5′-GCC​CAG​AAC​GCC​ATC​ACT​TG-3′ and sgRNA2: GGC​CAT​CAT​TGA​GTA​TCT​AG, were cloned into LentiCRISPR v2 vector (Addgene #52961). LentiCRISPR v2 control vector was used as the control vector. The third-generation lentiviral packaging system (Applied Biological Materials, cat. #52961) was used to prepare lentiviral particles in HEK293T cells. HEK293T cells were seeded at the density of 4 × 10^6^ per 10 cm cell culture dish followed by the addition of DNA mixtures. Cell culture medium containing viral particles was collected after 48-hour transfection. For the transduction, cells were seeded into six-well plates with a density of 1.5 to 2 × 10^5^ cells per well. Cells were incubated with 4 μg/mL of polybrene (Sigma, TR-1003-G) and the prepared lentiviral particles. Cell plates were centrifuged at 600 × *g* for 30 minutes and incubated for an additional 48 hours, followed by the addition of 1 μg/mL of puromycin for selection. *GSTZ1* knockout efficiency was confirmed using immunoblotting.

### Clonogenic assay

H1792 cells were seeded in six-well plates at the density of 1 × 10^4^ cells per well. Cell plates were placed on ice and gently washed with 1.5 mL of ice-cold phosphate-buffered saline (PBS). Cells were then fixed by adding 1.5 mL of ice-cold methanol for 10 minutes on ice. After removing methanol, 1.5 mL of 0.2% crystal violet solution (Sigma, HT90132) were added to each well and incubated for 30 minutes at room temperature. Cell plates were gently rinsed with distilled water and allowed to dry overnight. The bound crystal violet was extracted by adding 750 μL of methanol and incubated at room temperature for 60 minutes. Methanol solutions were transferred to a 96-well plate, and absorbance was measured at 540 nm on a M5 SpectraMax plate reader (Molecular Devices).

### Metabolomics sample preparation

H1792 cells were treated with nontargeting siRNA (siNT) and si*GSTZ1* for 24 hours, followed by an additional 72-hour treatment with sotorasib or DMSO. Cell pellets were washed three times with PBS and stored at −80°C until use. All procedures were carried out on ice to maintain sample integrity. To initiate protein precipitation, 500 μL of precooled 80% methanol extraction solvent were added to the samples. Following solvent addition, the samples were vortexed thoroughly and centrifuged at 18,800 × *g* (Microfuge 22R, Beckman Coulter) at 0°C for 10 minutes. To enhance metabolite extraction, the samples were then incubated in a −80°C freezer for 30 minutes before undergoing immediate centrifugation at 18,800 × *g* at 4°C for 10 minutes. The resulting supernatant was carefully transferred into a fresh microcentrifuge tube. Meanwhile, the protein pellet was resolubilized in aqueous 20 mmol/L HEPES buffer containing 8 mol/L urea for Bradford assays to quantify protein concentration. Dried metabolites were redissolved in 80% methanol containing 10% of the Metabolomics QC Kit, ensuring consistency across samples. A 2 μL aliquot of the Metabolomics QC Kit metabolite mixture was added to each sample to facilitate downstream analysis.

### Ultra-high-performance liquid chromatography–high-resolution mass spectrometry analysis of metabolomics samples

Ultra-high-performance liquid chromatography (UHPLC) coupled with high-resolution mass spectrometry was conducted using a Vanquish UHPLC system interfaced with a Q Exactive HF quadrupole Orbitrap mass spectrometer (Thermo Fisher Scientific). Chromatographic separation was achieved using an Atlantis Premier BEH Z-HILIC VanGuard FIT column (2.1 mm ID × 150 mm length, 2.5 μm particle size; Waters). Mobile phase A consisted of aqueous 10 mmol/L ammonium carbonate with 0.05% ammonium hydroxide, whereas mobile phase B was 100% acetonitrile. The gradient program was structured as follows: the initial condition was set at 80% B, followed by a linear decrease to 20% B over 13 minutes. The system was remained at 20% B for 2 minutes before swiftly returning to 80% B within 0.1 minutes, concluding with a 4.9-minute reequilibration period. The total runtime was 20 minutes, with a flow rate of 0.400 mL/minute.

The autosampler was maintained at 5°C, whereas the column temperature was stabilized at 30°C. A sample injection volume of 2 μL was used for both positive and negative ion mode electrospray ionizations. Full MS scans were performed separately in both ionization modes, detecting ions ranging from m/z 65 to m/z 900. Additionally, data-dependent acquisition was used for tandem MS (MS/MS) of pooled analyte samples, ensuring reliable metabolite identification and assignment confirmation.

### Metabolomics data analysis

Metabolite identification and quantification were performed using MZmine software (version 3.53), utilizing an in-house library for matching based on m/z and retention time (RT). A batch file streamlined the automation of multiple analytical modules, including centroid mass detection, ADAP chromatogram builder, and deconvolution via local minimum search. Key settings included a minimum group size of five scans and a group intensity threshold set to 1 × 10^4^. Smoothing parameters were adjusted to a value of five, and chromatographic thresholding was maintained at 95%. RT search parameters ranged from 0.05 to 5 minutes, ensuring precise peak detection. Additional refinements included isotopic peak grouping, peak alignment (weighted 75% for m/z and 25% for RT), duplicate peak filtering, and gap filling. Custom database searches were conducted with a tolerance of 10 ppm for m/z and 0.3 minutes for RT, ensuring robust metabolite identification. Adduct and complex searches, peak list filtering, and final peak list exports were executed seamlessly. Peak height values were extracted as CSV files for further processing using the iterative rank-order normalization method (31).

### Immunoblotting

Cells were harvested and washed with PBS and lysed in lysis buffer consisting of 0.2% NP-40, 50 mmol/L tris (pH 7.5), 5% glycerol, 1.5 mmol/L MgCl_2_, 100 mmol/L NaCl, and phosphatase (Sigma, P5726) and protease (Roche, cat. #11873580001) inhibitors for 30 minutes on ice. The Coomassie Plus (Bradford) protein assay (Thermo Fisher Scientific, 23236) was conducted for measuring concentrations of the resulting supernatants. Samples were prepared by adding 4× Laemmli sample buffer and heat-denaturing for 5 minutes. All samples were resolved by SDS–polyacrylamide gel electrophoresis and then transferred onto polyvinylidene fluoride (PVDF) membranes. Five percent nonfat milk was utilized for blocking of nonspecific binding. Blots were incubated with primary antibodies overnight at 4°C. For immunoblotting, primary antibodies were purchased from Cell Signaling Technology: phospho-AKT (S473; cat. #9271), phospho-p44/42 MAPK (ERK1/2; Thr202/Tyr204; cat. #4370), AKT (cat. #9272), phospho-AMPKα (Thr172; cat. #2535s), AMPKα (cat. #2532s), mTOR (cat. #2972s), and phospho-mTOR (Ser2448; cat. #5536s). DYKDDDDK tag monoclonal antibody (cat. #66008-4-Ig) and GSTZ1 (#14889-1-AP) were purchased from Proteintech. Anti-MAPK (ERK1/2; cat. #M5670) and anti-actin (cat. #A5441) antibodies were from Sigma. Secondary antibodies were from GE Healthcare: horseradish peroxidase–conjugated anti-rabbit (cat. #NA934) or anti-mouse (cat. #NA931) antibodies were applied and incubated with PVDF membranes for 1 hour. Images were obtained using the Odyssey Fc imaging system (LI-COR). Densitometry was analyzed using Image Studio Lite v6.0.

### ROS measurement and imaging

Cells were seeded at the density of 0.1 × 10^6^ cells per well in 12-well plates and maintained at 37°C overnight. Cells were treated with indicated drugs or siRNAs for 48 hours. After treatment, cells were washed with cell culture medium and incubated with 10 μmol/L 2,7-dichlorodihydrofluorescein diacetate (H_2_DCFH-DA) for 30 minutes in the dark. Cells were rinsed twice with PBS and immersed in 500 μL of PBS in each well. Representative fluorescent images were captured for each well using the GFP channel on a fluorescence microscope. After imaging, PBS was removed, and 200 μL of RIPA buffer were added to each well. The plates were incubated on ice for 15 minutes, after which the cell lysate was collected in 1.5-mL tubes and centrifuged at 21,130 × *g* for 10 minutes at 4°C. The supernatant (100 μL) was then transferred to a black 96-well plate, and fluorescence intensity was measured using a microplate reader with excitation at 485 nm and emission at 530 nm. In parallel, 2 μL of the supernatant were added to a clear 96-well plate containing 398 μL of 1× protein assay solution, and protein concentration was determined via the Bradford assay. Fluorescence intensities were normalized to their corresponding protein concentrations.

### Intracellular GSH measurement

Cells were seeded into six-well plates at the density of 0.5 × 10^6^ cells per well and incubated for 24 hours, followed by the treatment with si*GSTZ1* and siNTs for 48 hours. GSH levels were measured using the GSH assay kit (MedChemExpress, HY-K0311) according to the manufacturer’s protocol. Briefly, cells were rinsed twice with PBS, followed by lysis with 100 μL of the provided extraction buffer and sonication (5 pulses, 4 seconds on, 20 seconds off, and power output setting = 2). GSH standard solution was prepared as a series of concentrations (50, 25, 12.5, 6.25, 3.13, 1.57, and 0 μmol/L). To a 96-well plate, 20 μL of the GSH standard solution or sample were sequentially mixed with 65 μL of buffer solution, 60 μL of substrate working solution, and 60 μL of enzyme/coenzyme working solution. After 15 minutes incubation at room temperature, absorbance was recorded on the M5 SpectraMax plate reader (Molecular Devices) at 412 nm. GSH concentrations of the samples were calculated based on the GSH standard curves.

### Intracellular lactate measurement

Cells were seeded into six-well plates at the density of 0.5 × 10^6^ cells per well and incubated for 24 hours. Cells were treated with si*GSTZ1* and siNTs for additional 48 hours. Intracellular lactate concentrations were assayed using the L-Lacate Assay Kit (Cayman, 700510). Following 48-hour incubation, cells were washed with PBS and lysed using sonication (5 pulses, 4 seconds on, 20 seconds off, and power output setting = 2) in 50 μL of PBS supplemented with 1% NP-40. Fifty microliters of metaphosphoric acid assay reagent were added to cell lysis, followed by centrifugation at 10,000 × *g* for 10 minutes at 4°C. The precipitated proteins were solubilized in 8 mol/L urea supplemented with 5% 2-mercaptoethanol, followed by protein quantification and immunoblotting. The supernatant was transferred to 1.5 mL microcentrifuge tubes and neutralized by 5 μL of potassium carbonate solution. Samples were centrifuged at 10,000 × *g* for 5 minutes to remove precipitated salts, and the supernatant was transferred to clean tubes for measurement. Lactate standard solutions were prepared based on the manufacturer’s instruction. The 20 μL of standard solutions or samples were added to 96-well plates and then mixed with 100 μL of assay buffer, 20 μL of cofactor mixture, 20 μL of the lactate substrate, and 40 μL of enzyme mixture. Plates were covered and incubated for 20 minutes at room temperature. Absorbance was read at 535 nm. Lactate concentrations of samples were calculated using the equation calculated from the lactate standard curve.

### *GSTZ1* overexpression

H1792 cells were seeded in six-well plates at the density of 0.5 × 10^6^ cells per well. To each well, 3 μg of *GSTZ1* expression plasmid (SinoBiological, HG14237-NF) or the negative control vector (SinoBiological, CV016) were mixed with 9 μL of lipofectamine 2000 reagent (Invitrogen, 11668) in Opti-MEM (Thermo Fisher Scientific, 31985062) and incubated for 5 minutes. DNA–lipid complex was added to cells and incubated for 48 hours. Hygromycin was added to cells at the final concentration of 200 μg/mL. Cells were passaged every 3 to 4 days and maintained in the presence of hygromycin for 12 days.

### Data mining and bioinformatic analysis

DepMap interactive portal was utilized to exam the correlation of metabolite abundance and gene expression levels with *GSTZ1* or *KRAS* gene dependency across diverse lung cancer cell lines. Pathway and metabolite enrichment analysis was conducted using Enrichr (32). The Kaplan–Meier plotter platform was used to evaluate the association between *GSTZ1* gene expression and overall survival (OS), postprogression survival (PPS), and first progression (FP) in patients with lung cancer. The GSE31210 dataset was selected for analysis, and *GSTZ1* expression was queried using the Affymetrix probe ID 209531_at. The auto-select best cutoff and censored at threshold options were selected, whereas all remaining parameters are defined as default values.

### Statistical analysis

Statistical analyses were conducted using GraphPad Prism software (GraphPad Software, version 10.0). Data are presented as the mean ± standard deviation (SD). All statistical tests were performed using the complete set of observed experimental data and were two-sided, with *P* values of <0.05 considered statistically significant. Specific statistical parameters and methods are detailed in the corresponding figures and figure legends. For comparisons between the two groups, Welch *t* test was applied. In the context of multiple comparisons, one-way ANOVA followed by Tukey *post hoc* test was applied.

## Results

### GSTZ1 is upregulated in *KRAS*^*G12C*^-mutant NSCLC and promotes resistance to KRAS^G12C^ inhibitors

**Figure 1.**
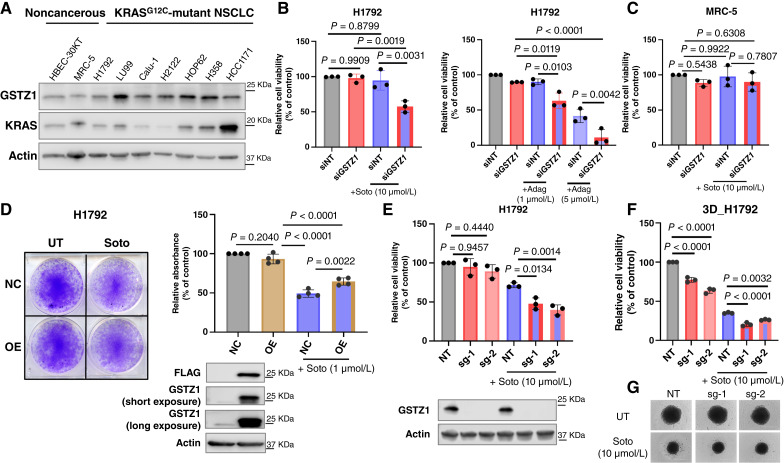
GSTZ1 modulates the efficacy of KRAS^G12C^ inhibitors in *KRAS*^*G12C*^-mutant NSCLC. **A,** Immunoblot analysis of GSTZ1 protein levels in noncancerous lung and *KRAS*^*G12C*^-mutant NSCLC cell lines. **B** and **C,** Relative cell viability of H1792 and MRC-5 cells transfected with siNT or si*GSTZ1* for 24 hours, followed by the treatment with sotorasib (Soto) or adagrasib (Adag) at indicated concentrations for additional 72 hours. *N* = 3. **D,** Crystal violet staining (left) and absorbance quantification (right) in H1792 cells with *GSTZ1* overexpression (OE) or nontargeting control (NC) treated with Soto. *GSTZ1* overexpression was confirmed by immunoblotting. *N* = 4. **E,** Viability of H1792 cells upon *GSTZ1* knockout using two individual guide RNAs, sg-1and sg-2, with or without Soto treatment for 96 hours in 2D (*N* = 3). GSTZ1 protein levels were validated by immunoblotting. **F,** Cell viability of H1792 cells with NT and *GSTZ1* knockout in 3D culture enabled using 96-well spheroid microplates. Soto (10 μmol/L) was incubated with spheroids for 72 hours after 48-hour spheroid formation. **G,** Representative brightfield images of 3D H1792 spheroids shown at scale bars of 400 μm. Cell viability was measured using 3D CellTiter-Glo luminescent cell viability assay. *N* = 3. Data represent the mean ± SD. Statistical significance was determined by one-way ANOVA with Tukey multiple comparisons test.

We recently reported that *GSTZ1* overexpression is significantly related to poor prognosis of patients with NSCLC and that *GSTZ1* displays codependency with *KRAS* in a subset of drug-insensitive NSCLC cell lines, including H1792 and H2122 (15). Here, we expanded our study by examining GSTZ1 protein levels across a panel of *KRAS*^*G12C*^-mutant–positive (+) NSCLC cell lines and noncancerous lung cell line controls. Immunoblot analysis revealed elevated GSTZ1 levels in *KRAS*^*G12C*^+ lines compared with two noncancerous lung cell lines, the HBEC30KT bronchial epithelial cell line and the lung fibroblast cell line MRC-5 (Fig. 1A). Silencing *GSTZ1* using siRNAs did not affect basal viability but significantly enhanced sensitivity to sotorasib or adagrasib in H1792 NSCLC cells, while sparing MRC-5 fibroblasts (Fig. 1B and C). This sensitization effect was recapitulated in KRAS^G12C^ inhibitor–insensitive LU99, Calu-1, H2122, and HOP62 and KRAS^G12C^ inhibitor–sensitive HCC1171 and H358 cells (Supplementary Fig. S1A–S1G). To examine whether the sensitization effect by GSTZ1 targeting is KRAS^G12C^-specific, we tested the combination of GSTZ1 knockdown and the pan-RAS inhibitor RMC-7977 in NSCLC H460 cells harboring KRAS Q61H mutation (Supplementary Fig. S1H) and PC-9 cells harboring EGFR Ex19Del mutation (Supplementary Fig. S1I). Similarly, we observed sensitization effect by knocking down GSTZ1 in both cell lines, indicating that GSTZ1 targeting can produce a broader sensitization, including but not limited to *KRAS* mutations. Conversely, forced overexpression of *GSTZ1* dampened sotorasib response in H1792 cells in clonogenic survival assays, confirming a modulatory role of GSTZ1 in the cell response to KRAS^G12C^ inhibitors (Fig. 1D). Notably, CRISPR-mediated *GSTZ1* knockout augmented the antiproliferative effects of KRAS inhibitors in both 2D (Fig. 1E; Supplementary Fig. S1J) and 3D cell cultures (Fig. 1F and G; Supplementary Fig. S1K and S1L). Moreover, we found that *GSTZ1* overexpression not only significantly correlated with sotorasib activity across lung cancer cell lines (Supplementary Fig. S1M) but also with OS, PPS, and FP of patients with lung cancers (Supplementary Fig. S1N). In conclusion, these results confirm that *GSTZ1* is selectively upregulated in a panel of KRAS inhibitor–insensitive *KRAS*^*G12C*^ + NSCLC cell lines and modulates cell sensitivity to KRAS^G12C^-targeted inhibition.

### *GSTZ1* knockdown reprograms cell metabolism by disrupting redox homeostasis and glycolysis

**Figure 2.**
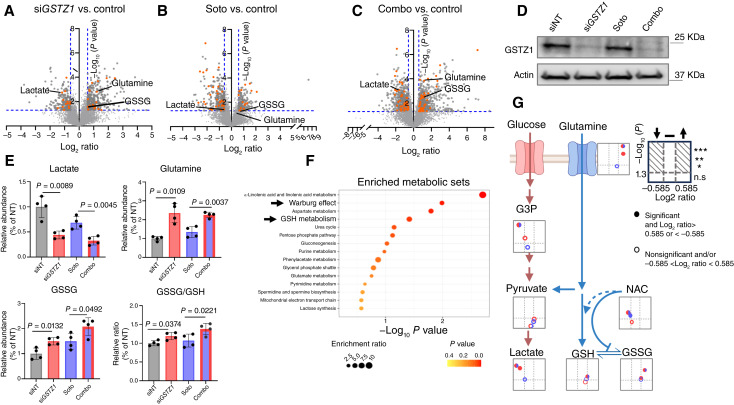
*GSTZ1* depletion induces metabolic alterations in *KRAS*^*G12C*^-mutant NSCLC. **A,** Effects of *GSTZ1* silencing (red circles), sotorasib (Soto) treatment (blue circles) or their combination (red/blue semicircles) on glycolysis and glutamine metabolism. Solid circles in volcano blots, in contrast to empty circles, represent significant changes in signals that pass both the significance and log_2_ ratio cutoffs, shown by dashed lines. Lines and arrows denote direct or indirect interactions. The ratio reflects the metabolite signal of each treatment compared with the siNT control. Glyceraldehyde 3-phosphate is abbreviated as G3P. **B,** Immunoblots of GSTZ1 and actin. **C–E,** Volcano plots showing significantly altered annotated metabolites (orange) in H1792 cells following the treatment with si*GSTZ1*, Soto (1 μmol/L), or combination treatment vs. control. Significance cutoffs were applied: log_2_ ratio ±0.585 and *P* < 0.05, and selected metabolites were highlighted. **F,** Bar plots of relative metabolite abundance of lactate, glutamine, GSSG, and the ratio of GSSG to GSH. Lactate, glutamine, GSSG, and GSH signals were extracted from **C–E**. **G,** Enriched metabolic pathways based on significantly altered metabolites with GSH and glycolysis pathways highlighted. Statistical analysis was conducted using Welch *t* test. Mean ± SD shown for four replicates (*N* = 4). *, *P* < 0.05; **, *P* < 0.01; ***, *P* < 0.001; n.s., not significant.

To investigate the effect of *GSTZ1* targeting on cell metabolism, we conducted untargeted metabolomics in H1792 cells treated with si*GSTZ1*, sotorasib, or their combination for 96 hours (Supplementary Fig. S2A). The metabolomics results showed significant metabolic alterations in glycolysis and GSH metabolism upon KRAS^G12C^ inhibition and/or *GSTZ1* knockdown (Fig. 2A–C; Supplementary Table S2). GSTZ1 protein reduction by si*GSTZ1* was confirmed by Western blotting (Fig. 2D). We observed significant decreases in lactate, increases in glutamine, increases in oxidized GSH (GSSG), and the enhancement of the ratio of GSSG to GSH (Fig. 2E). The GSH amount was not significantly changed upon *GSTZ1* knockdown when compared with siNT treatment (Supplementary Fig. S2B and S2C). Interestingly, significant increases in glutamine levels were noted with *GSTZ1* knockdown (Fig. 2E). This is likely a compensatory mechanism for the suppressed antioxidant defense and the decreased glycolytic activity, as glutamine serves as an energy source and a precursor for the synthesis of the major antioxidant GSH (33). Metabolic pathway enrichment analysis indicated significant enrichment of the Warburg effect and GSH metabolism (Fig. 2F), suggesting impaired glycolytic flux and oxidative imbalance after *GSTZ1* knockdown. In addition, knockdown of *GSTZ1* altered a broad array of metabolites involved in purine, pyrimidine, glycolysis, redox, and fatty acid pathways (Supplementary Fig. S2B). Our study identified increased levels of eicosapentaenoic acid (EPA; Supplementary Fig. S2D) and docosahexaenoic acid (DHA; Supplementary Fig. S2E), both of which are omega-3 fatty acids, indicating an additional role of *GSTZ1* in unsaturated fatty acid metabolism. To study whether there are distinct metabolic phenotypes that are responsible for the sensitization by GSTZ1 targeting, we categorized cell lines based on their degree of sensitization to GSTZ1 knockdown in combination with KRAS inhibition by using the cell viability fold change of the combination relative to sotorasib treatment alone (Supplementary Fig. S2F). H1792, H2122, and H460 were classified as sensitive, with all remaining lines designated as less sensitive. Differentially expressed genes between these two groups were identified using DepMap gene expression data (Supplementary Table S3) and subjected to pathway and metabolite enrichment analyses (Supplementary Fig. S2G). The sensitive group exhibited significant enrichment of GSH-associated pathways and genes linked to GSH and pyruvic acid metabolism (Supplementary Fig. S2H). Representative expression profiles of key genes within these pathways are shown in Supplementary Fig. S2I. Consistent with this, glutamine abundance positively correlated with both *GSTZ1* and *KRAS* gene dependency (Supplementary Fig. S2J). Collectively, these findings align with our mechanistic model in which GSTZ1 knockdown sensitizes *KRAS*-mutant cells by perturbing GSH metabolism and glycolytic flux. Taken together, *GSTZ1* targeting can rewire cellular metabolism, thereby leading to glycolysis pathway suppression and redox imbalance (Fig. 2G).

### GSTZ1 maintains GSH homeostasis and restrains ROS accumulation

**Figure 3.**
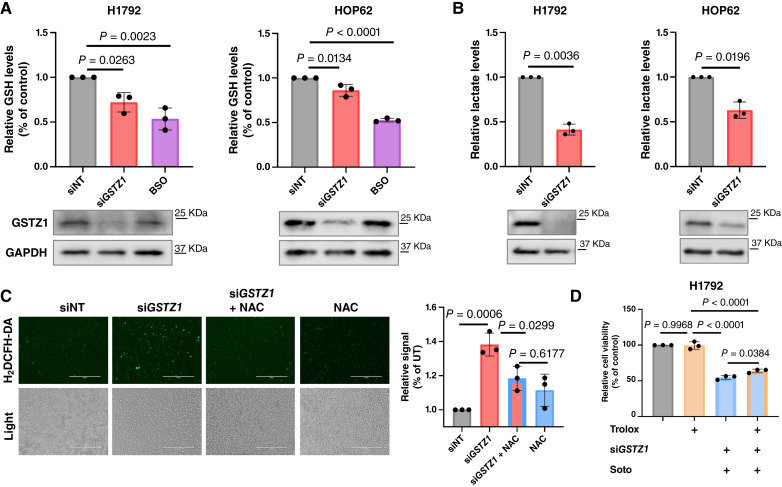
*GSTZ1* knockdown impairs redox balance and sensitizes NSCLC cells to oxidative stress. **A,** Relative GSH and (**B**) lactate levels in H1792 and HOP62 cells following si*GSTZ1* or BSO (500 μmol/L) treatment for 48 hours. *GSTZ1* knockdown was confirmed by immunoblotting. *N* = 3. **C,** Images of ROS signals via H_2_DCFH-DA fluorescence in H1792 cells following treatment with si*GSTZ1*, N-acetyl cysteine (NAC, 2 mmol/L), and their combination for 48 hours. H_2_DCFH-DA fluorescence intensity was measured using a microplate reader at the excitation 485 nm and emission 530 nm. *N* = 3. **D,** Viability of H1792 cells treated with si*GSTZ1*, sotorasib (Soto; 1 μmol/L), and the antioxidant Trolox (50 μmol/L) for 96 hours. Cell viability was measured using CellTiter-Glo luminescent cell viability assay. *N* = 3. Welch *t* test was applied for the comparison of lactate levels in H1792 and HOP62 cells. Other data were analyzed by one-way ANOVA with Tukey multiple comparisons test. Data represent the mean ± SD.

GSH metabolism and glycolysis play an essential role in sustaining *KRAS* mutation–driven tumorigenesis (34, 35). In hepatocellular carcinoma, *GSTZ1* targeting was reported to deplete the intracellular GSH pool and induce oxidative stress by increasing ROS levels via nonenzymatic bypass reactions (2, 12, 14). Therefore, we examined intracellular GSH levels in multiple *KRAS*^*G12C*^-mutant cell lines treated with si*GSTZ1* for 48 hours. Similar to the effects of buthionine sulfoximine (BSO), a GSH synthesis inhibitor, *GSTZ1* knockdown led to marked reductions in GSH in H1792, LU99, and H358 (Fig. 3A; Supplementary Fig. S3A), whereas HOP62 and H2122 exhibited smaller decreases. Lactate is a key marker of glycolysis in cancer cells, reflecting the Warburg effect that cancer cells heavily rely on for energy production and cell proliferation. Upon silencing of *GSTZ1*, we found significant decreases in the levels of intracellular lactate in H1792, HOP62, and H358 cells (Fig. 3B; Supplementary Fig. S3B). As we detected lower levels of GSH upon *GSTZ1* targeting and GSH depletion can lead to oxidative stress by increasing ROS levels, we measured the ROS levels using H_2_DCFH-DA fluorescence. As expected, ROS levels significantly increased upon *GSTZ1* silencing and could be attenuated by the antioxidant N-acetyl cysteine (Fig. 3C; Supplementary Fig. S3C), which agrees with reported GSH reduction and ROS elevation following *GSTZ1* silencing in liver cancer (13). Importantly, the reduced cell viability upon *GSTZ1* depletion and sotorasib treatment was partially rescued by cotreatment with the antioxidant trolox in H1792 and LU99 cells (Fig. 3D; Supplementary Fig. S3D). In summary, GSTZ1 safeguards cancer cells by preserving GSH levels and preventing oxidative stress, thereby limiting ROS-induced cytotoxicity.

### Targeting GSH and glycolytic pathways enhances KRAS^G12C^ inhibitor efficacy

**Figure 4.**
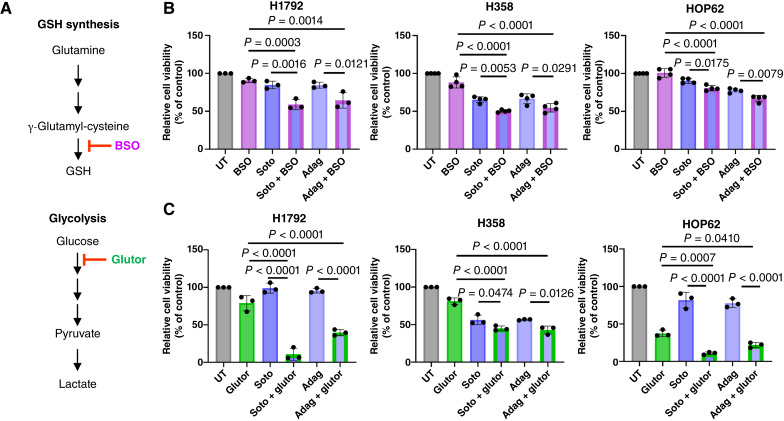
Disruption of GSH and glycolysis pathways enhances KRAS^G12C^ inhibitor efficacy. **A,** Scheme of mechanisms of action of BSO and glutor. **B,** Cell viability of H1792 (*N* = 3), H358 (*N* = 4), and HOP62 cells (*N* = 4) treated with BSO (500 μmol/L) or (**C**) glutor (50 nmol/L) alone or in combination with sotorasib (Soto; 10 μmol/L) or adagrasib (Adag; 1 μmol/L) for 96 hours. *N* = 3. Cell viability was measured using CellTiter-Glo luminescent cell viability assay. Data represent the mean ± SD (*N* = 3) and were analyzed by one-way ANOVA followed by Tukey *post hoc* test.

To determine whether glycolytic inhibition and GSH depletion are mechanistically associated with KRAS inhibitor sensitivity, we combined KRAS^G12C^ inhibitors with small-molecule inhibitors targeting GSH synthesis and glycolysis, respectively (Fig. 4A). Cotreatment with BSO significantly amplified sotorasib- and adagrasib-mediated cytotoxicity in H1792, H358, HOP62, LU99, and H2122 cells (Fig. 4B; Supplementary Fig. S4A and S4B). Glutor, which inhibits glycolysis upfront by targeting glucose transporters, also remarkably sensitized H1792, H358, and HOP62 cells to KRAS^G12C^ inhibitors (Fig. 4C). This sensitization effect was somewhat less pronounced in LU99 and H2122 cells (Supplementary Fig. S4C and S4D), suggesting a cell type–specific response to the mechanism via glycolytic inhibition. In summary, inhibiting GSH synthesis and glycolysis potentiates the efficacy of KRAS^G12C^ inhibitors in *KRAS*^*G12C*^-mutant NSCLC cells.

### *GSTZ1* targeting modulates AMPK–AKT signaling

**Figure 5.**
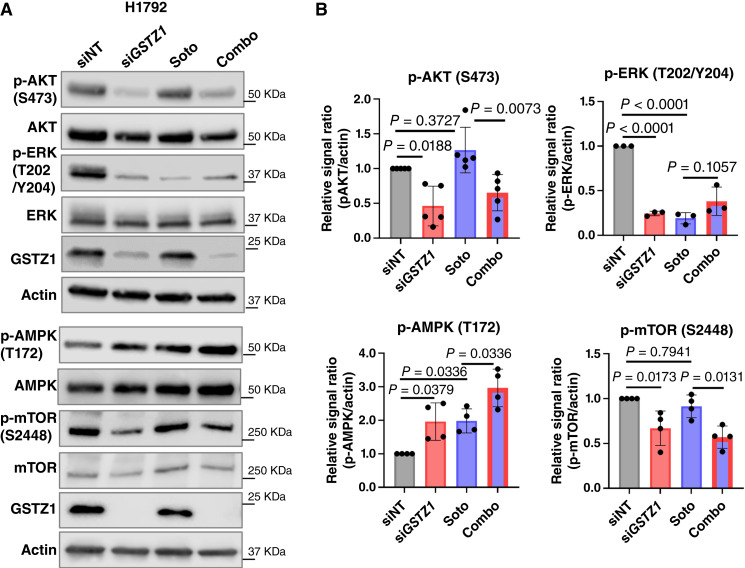
GSTZ1 inhibition enhances AMPK phosphorylation and suppresses survival signaling. **A,** Immunoblots of H1792 lysates showing levels of phosphorylated and total AKT, ERK, AMPK, mTOR, and GSTZ1 following si*GSTZ1*, sotorasib (Soto; 1 μmol/L), and combined treatment for 96 hours. **B,** Quantification of phospho-protein signals relative to actin: p-AKT (S473, *N* = 5), p-ERK (T202/Y204, *N* = 3), p-mTOR (S2448, *N* = 4), and p-AMPK (T172, *N* = 4). Data represent the mean ± SD and were analyzed by one-way ANOVA with Tukey *post hoc* test.

AMPK acts as a sensor for energy and oxidative stress and is activated through increased phosphorylation at T172 when cells are under oxidative stress and have reduced glycolytic activity (19–21). More importantly, AMPK negatively regulates key downstream signaling effectors such as AKT and mTOR, which are essential for the survival of *KRAS*-mutant cancer cells and for modulating sensitivity to KRAS inhibitors. We previously reported that *GSTZ1* knockdown affected proteome-wide protein tyrosine phosphorylation (15). We now observed that *GSTZ1* knockdown also significantly reduced the phosphorylation of ERK and AKT, two critical kinases in cell survival and drug resistance to KRAS^G12C^ inhibitors (36). Notably, the reduction of AKT phosphorylation was not observed with sotorasib treatment alone in H1792 cells (Fig. 5A and B), suggesting that GSTZ1 modulates AKT signaling irrespective of KRAS. We also found increased phosphorylation of AMPK and decreased phosphorylation of mTOR (Fig. 5A and B). As these phosphorylation sites reflect activation status of both kinases, this indicates activation of the tumor-suppressor AMPK and inhibition of protumorigenic mTOR. Moreover, GSH depletion by BSO and glycolytic inhibition by glutor increased the phosphorylation of AMPK (Supplementary Fig. S5A), recapitulating the effect of *GSTZ1* targeting on glycolysis and GSH metabolism. This observation aligns with the established impact to AMPK signaling due to oxidative stress and glycolytic inhibition (37, 38). Increased AMPK and decreased AKT upon *GSTZ1* targeting are consistently observed in additional LU99 and HOP62 cells (Supplementary Fig. S5B and S5C). ERK phosphorylation remained suppressed during sotorasib treatment as well (Supplementary Fig. S5D and S5E). Taken together, *GSTZ1* targeting impairs survival signaling by AKT and ERK and stimulates AMPK stress response signaling.

### Pharmacologic modulation of the metabolic stress sensor AMPK sensitizes *KRAS*^*G12C*^-mutant NSCLC cells

**Figure 6.**
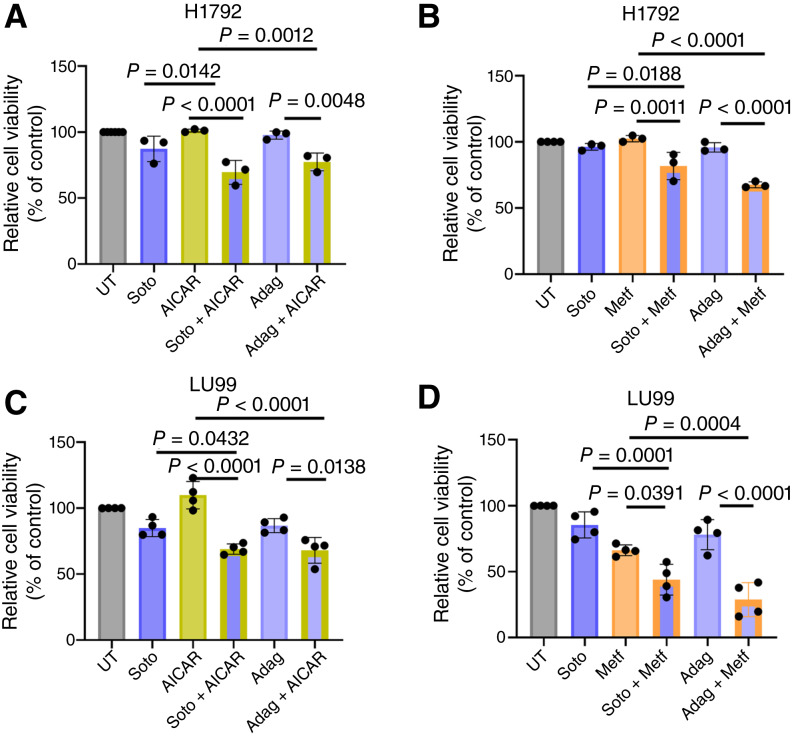
Activation of AMPK signaling enhances the efficacy of KRAS^G12C^ inhibition. **A** and **B,** Relative viability of H1792 cells treated with sotorasib (Soto; 10 μmol/L) or adagrasib (Adag; 1 μmol/L) alone or in combination with AMPK activators acadesine (AICAR, 0.5 mmol/L) or metformin (Metf, 0.5 mmol/L) for 96 hours. *N* = 3. **C** and **D,** Relative viability of LU99 cells treated with Soto (10 μmol/L) or Adag (1 μmol/L) alone or in combination with AICAR (0.5 mmol/L) or Metf (0.5 mmol/L) for 96 hours. *N* = 4. Data are presented as the mean ± SD (*N *= 3) and were analyzed using one-way ANOVA with Tukey *post hoc* test.

To investigate whether these mechanisms contribute to the cell sensitization to KRAS^G12C^ inhibitors, we used two pharmacologic AMPK activators. Acadesine (AICAR) is a cell-permeable nucleoside, and its intracellularly converted AICAR monophosphate metabolite binds the regulatory γ subunit to activate AMPK (39). Metformin is a well-known AMPK activator acting by the inhibition of complex 1 of the mitochondrial respiratory chain (40) and promoted formation of the AMPK αβγ complex at relatively low doses (41). Our results show that AICAR (Fig. 6A) and metformin (Fig. 6B) significantly enhanced cell response to both sotorasib and adagrasib in H1792 cells. Similarly, these cotreatments increased the cytotoxicity of sotorasib and adagrasib in additional LU99 cells (Fig. 6C and D). These results indicate that pharmacologic activation of AMPK mimics the effect of *GSTZ1* knockdown on cell sensitization, thus highlighting the role of AMPK in mediating therapy response.

## Discussion

This study highlights a novel therapeutic avenue in *KRAS*-mutant NSCLC by uncovering the pivotal role of GSTZ1 in metabolic adaptation, drug efficacy improvement, and cell survival. Our findings demonstrate that *GSTZ1* expression confers resistance to KRAS^G12C^ inhibitors, including sotorasib and adagrasib, and that its depletion significantly enhances activity of these inhibitors in both drug-sensitive and drug-insensitive NSCLC cells. Mechanistically, *GSTZ1* loss induced profound metabolic rewiring, characterized by diminished glycolytic flux and GSH metabolism, as evidenced by reductions in lactate and GSH levels and increases in GSSG and glutamine. These shifts generate a state of energetic and oxidative stress that seems to impair cancer cell viability and enhance vulnerability to targeted therapy. This finding also aligns with prior studies linking oxidative imbalance and impaired glycolysis with increased *KRAS*-mutant targeting efficacy (34, 42).

Importantly, our study further elucidates the signaling consequences of *GSTZ1* targeting. *GSTZ1* knockdown led to substantial alterations in protein phosphorylation, as evidenced by our previous publication showing the effect of *GSTZ1* targeting on the tyrosine phosphoproteome (15). We now furthermore observed pronounced effects on suppression of AKT and ERK activity in multiple *KRAS*^*G12C*^ + NSCLC cell lines, which are key drivers of survival and therapeutic resistance in *KRAS*-driven tumors (36, 43). Concomitantly, we observed activation of the AMPK pathway and inhibition of phosphorylation of mTOR, which are pathways closely associated with redox and energetic stress and subsequent antiproliferative responses (44, 45). AMPK activation is considered antitumorigenic and reported to increase cell sensitivity in *KRAS*^*G12C*^-mutant NSCLC H23 cells (44, 45). *KRAS*^*G12C*^ mutation was reported to specifically boost the levels of carnitine and carnitine derivatives, which are involved in the oxidation of fatty acids (46). Consistently, our metabolomics study also showed decreased carnitine and relevant derivatives upon KRAS^G12C^ inhibition by sotorasib. Additionally, a previous study revealed that the ablation of *GSTZ1* resulted in enhanced lipid peroxidation and the induction of ferroptosis in hepatocellular carcinoma (13). Our study observed elevated levels of two omega-3 fatty acids, EPA, and DHA, which have demonstrated antiproliferative effects against lung cancer cells (47). This finding suggests that GSTZ1 may enhance the sensitivity of lung cancer cells to drug treatment by multiple mechanisms, including modulating redox stress response, glycolysis, and potentially fatty acid metabolism. The convergence of metabolic and signaling disruptions presents GSTZ1 as a dual-function target that affects both upstream metabolic circuits and downstream survival pathways. Moreover, GSH synthesis or glycolytic inhibitors recapitulated the metabolic stress phenotype, such as increased phosphorylation of AMPK and enhanced KRAS^G12C^ inhibitor sensitivity in multiple cell lines. LU99 and H2122 cells were sensitized to a lesser extent by the cotreatment of glutor and KRAS^G12C^ inhibitors, indicating a cell type–specific response to the dual inhibition of glycolysis and mutant KRAS. This discrepancy may be associated with differential adaptive signaling responses to KRAS^G12C^ inhibitors (28) or metabolic adaptation capabilities to compensate for the induced stresses. For instance, LU99 cells have a much stronger ability to generate an important end product pyruvate of glycolysis from alanine through the highly expressed alanine aminotransferase (GPT2; ref. 48), which may mitigate the inhibitory effect of glycolysis by glutor. From the perspective of therapeutic development, cotargeting glycolysis and/or redox homeostasis along with oncogenic *KRAS* mutants becomes an emerging and attractive treatment approach, which is rationalized based on the close interplay between KRAS protein family, cancer cell glycolysis, and redox balance (5). These mechanisms have been widely explored in various *in vitro* and *in vivo* cancer models, including but not limited to KRAS (5, 49–52). There are ongoing clinical trials involving these mechanisms for the treatment of *KRAS*-mutant cancers (NCT06336902 and NCT04250545). These results support the feasibility of combining metabolic modulation with targeted therapies to improve the initial drug response in *KRAS*-mutant NSCLC. Several recently developed modalities targeting *KRAS* mutations, such as a pan-KRAS inhibitor BI-2865 (53) and a RAS(ON) inhibitor daraxonrasib (54), can be potentially selected to combine with *GSTZ1* targeting, and such combinations warrant future investigation in drug-insensitive *KRAS*-mutant cancer models.

In conclusion, GSH metabolism and glycolysis play an important role in sustaining *KRAS* mutant–driven tumorigenesis, and our results establish GSTZ1 as a key metabolic and signaling node that regulates GSH levels and glycolytic activity to cooperate with KRAS functionality. This discovery positions GSTZ1 as a novel target for *KRAS*-mutant NSCLC survival and therapeutic response. Targeting *GSTZ1* not only disrupts glycolytic and redox homeostasis but also represses survival signaling pathways such as AKT and mTOR while stimulating tumor-suppressive AMPK signaling. Given the similar regulation of cell metabolism and signaling effect of GST isoenzyme family (11, 55), it becomes intriguing to investigate whether other GSTs are involved in the modulation of the response to the *GSTZ1* targeting and/or KRAS-targeted inhibitors and whether multitargeting GSTs and *KRAS* mutants elicits more effective therapeutic outcomes as the multitargeting strategy usually yields beneficial effects in cancer (52, 56, 57). These findings will lay the groundwork for rationally designed combination strategies that exploit metabolic vulnerabilities to enhance the efficacy of KRAS-targeted therapies.

This study has limitations. The use of *in vitro* models, such as 2D and 3D cultures, may not fully recapitulate the complexity of the tumor microenvironment and immune interactions *in vivo*. Although metabolomic profiling provided valuable mechanistic insights, further validation in animal models and clinical specimens is required to establish the translational potential of GSTZ1-targeted interventions. Additionally, the present data do not unambiguously identify mitochondrial ROS or other compartment-specific ROS species as the primary mediators that are mechanistically relevant to observed GSTZ1-targeting effects, and the precise mechanism by which GSTZ1 regulates AMPK, ERK, and AKT signaling independent of KRAS remain to be fully elucidated. Moreover, the therapeutic effect of *GSTZ1* targeting requires the development of selective pharmacologic inhibitors of GSTZ1, which may possibly elicit different effects from RNAi-mediated target depletion.

## Supplementary Material

Table S1 Table S1 includes all key resources used in this study.

Table S2 Table S2 shows the metabolomic changes for H1792 cells upon treatment with siGSTZ1, sotorasib, and their combination.

Table S3 Table S3 shows DepMap gene expression signatures stratifying lung cancer lines by sensitivity to combined GSTZ1 and KRAS G12C co-targeting.

Figure S1 Figure S1 shows the effect of GSTZ1 silencing or knockout on cell viability and sensitivity to KRAS G12C inhibitors across KRAS G12C NSCLC lines in 2D and 3D, and their corresponding immunoblots. It also includes DepMap correlation and survival analysis.

Figure S2 Figure S2 shows the metabolomics workflow and the effect of GSTZ1 knockdown with or without sotorasib on glutathione, glycolytic, and lipid pathways with enriched pathway annotations. DepMap-based gene expression analysis shows correlation of GSH and GSH metabolism with GSTZ1-targeting effect.

Figure S3 Figure S3 shows the effect of GSTZ1 silencing, along with BSO or glutor, on GSH, lactate, and ROS levels, antioxidant rescue and cell viability.

Figure S4 Figure S4 shows the effect of pharmacologic inhibition of glutathione synthesis with BSO or glycolysis with glutor on KRAS G12C inhibitor cytotoxicity in LU99 and H2122 cells.

Figure S5 Figure S5 shows the effect of GSTZ1 loss or combined metabolic inhibition on p-AMPK, p-AKT and p-ERK signaling across cell lines.

## Data Availability

The raw metabolomics data generated in this study have been deposited in the MetaboLights repository with the dataset identifier/accession number MTBLS12791 (https://www.ebi.ac.uk/metabolights/editor/MTBLS12791/overview). All other data are available in the main article, supplemental files, or upon request to the corresponding author.
