# Supplementary material for: Targeting GSTZ1 Sensitizes KRASG12C-Mutant Lung Cancer Cells by Overcoming Glutathione and Glycolysis Pathway Rewiring
Source: Cancer Res Commun. 2026 Jun 11;6(6):1376–87. doi: 10.1158/2767-9764.CRC-25-0698 (PMC13254912; doi:10.1158/2767-9764.CRC-25-0698)
Supplement: Table S1 — includes all key resources used in this study. [file crc-25-0698_table_s1_suppst1.docx]

Table S1. Key Resource Table

| **Reagent/Resource** | **Reference or Source** | **Identifier or Catalog Number** |
| --- | --- | --- |
| **Experimental Models** |  |  |
| HBEC-30KT | (27), received on 2021/04/27 | Dr. John Minna’s laboratory at UT Southwestern Medical Center Dallas  ATCC: CRL-4051 |
| H1792 | Lung Cancer Center of Excellence at Moffitt  (28), received on 2021/12/23 | RRID:CVCL_1495 |
| H358 | Lung Cancer Center of Excellence at Moffitt  (28), received on 2021/11/16 | RRID:CVCL_1559 |
| LU99 | Lung Cancer Center of Excellence at Moffitt  (28), received on 2023/09/05 | RRID:CVCL_3015 |
| H2122 | Lung Cancer Center of Excellence at Moffitt  (28), received on 2023/08/23 | RRID:CVCL_1531 |
| HOP62 | Lung Cancer Center of Excellence at Moffitt  (28), received on 2022/08/24 | RRID:CVCL_1285 |
| Calu-1 | Lung Cancer Center of Excellence at Moffitt  (28), received on 2022/01/03 | RRID:CVCL_0608 |
| HCC1171 | Lung Cancer Center of Excellence at Moffitt  (29), received on 2023/09/05 | RRID:CVCL_5126 |
| MRC-5 | Lung Cancer Center of Excellence at Moffitt  (30), received on 2020/08/02 | RRID:CVCL_0440 |
| **Recombinant DNA** |  |  |
| lentiCRISPR v2 | Addgene | Plasmid #52961 |
| lentiCRISPR v2-sgControl | Addgene | Plasmid #125836 |
| Human GSTZ1 Gene ORF  cDNA clone expression  plasmid, N-Flag tag | SinoBiological | Cat# HG14237-NF |
| pCMV3-N-FLAG Negative Control Vector (N-terminal FLAG-tagged) | SinoBiological | Cat# CV016 |
| **Antibodies** |  |  |
| Monoclonal Anti-GAPDH antibody produced in mouse | GE Healthcare | Cat# G8795, RRID: AB_1078991 |
| Mouse IgG, HRP-linked whole Ab | GE Healthcare | Cat# NA931, RRID: AB_772210 |
| Phospho-p44/42 MAPK (Erk1/2) (Thr202/Tyr204) monoclonal antibody antibody | Cell Signaling | Cat# 4370, RRID: AB_2315112 |
| Phospho-Akt (Ser473) polyclonal Antibody | Cell Signaling | Cat# 9271, RRID: AB_329825 |
| Akt polyclonal Antibody | Cell Signaling | Cat# 9272, RRID: AB_329827 |
| Anti-MAP Kinase (ERK-1, ERK-2) antibody produced in rabbit | Sigma | Cat# M5670, RRID: AB_477216 |
| Rabbit IgG, HRP-linked whole Ab | GE Healthcare | Cat# NA934, RRID: AB_772206 |
| GSTZ1 Polyclonal antibody | Proteintech | Cat# 14889-1-AP, RRID: AB_2116363 |
| Phospho-AMPKα (Thr172) (40H9) Rabbit mAb | Cell Signaling | Cat# 2535s, RRID: AB_331250 |
| AMPKα Antibody | Cell Signaling | Cat# 2532S, RRID:AB_330331 |
| DYKDDDDK tag Monoclonal antibody (Binds to FLAG® tag epitope) | Proteintech | Cat# 66008-4-Ig, RRID:AB_2918475 |
| mTOR Antibody | Cell Signaling | Cat# 2972S, RRID:AB_330978 |
| Phospho-mTOR (Ser2448) (D9C2) XP® | Cell Signaling | Cat# 5536S, RRID:AB_10691552 |
| Anti-β-Actin (ACTB) Antibody | Sigma | Cat# A5441, RRID:AB_476744 |
| **Oligonucleotides and other sequence-based reagents** |  |  |
| ON TARGET plus non-targeting siRNA pool | Dharmacon | Cat# D-001810-10-20 |
| ON-TARGETplus Human GSTZ1 (2954) siRNA - SMARTpool | Dharmacon | Cat# L-011290-00-0010 |
| GSTZ1‐sgRNA1 seq: GCCCAGAACGCCATCACTTG | (13) |  |
| GSTZ1‐sgRNA2 seq: GGCCATCATTGAGTATCTAG | This study |  |
| **Chemicals, Enzymes and other reagents** |  |  |
| Sotorasib (AMG-510) | ChemieTek | Cat# CT-AMG510 |
| Adagrasib | MedChemExpress | Cat# HY-130149 |
| DL-Buthionine-(S,R)-sulfoximine (BSO) | MedChemExpress | Cat# HY-106376 |
| Glutor | Sigma | Cat# SML2765 |
| Lipofectamine^TM^ RNAiMAX | ThermoFisher | Cat# 13778150 |
| Opti-MEM^TM^ | ThermoFisher | Cat# 31985062 |
| DMSO used in cell culture | Sigma | Cat# D2438 |
| Fetal bovine serum | Sigma | Cat# F2442 |
| RPMI media | ThermoFisher | Cat# SH30027FS |
| CellTiter-Glo_ Luminescent Cell Viability Assay | Promega | Cat# G7573 |
| Trolox | Cayman Chemical | Cat# 10011659 |
| Acadesine (AICAR) | MedChemExpress | Cat# HY-13417 |
| Metformin | MedChemExpress | Cat# HY-B0627 |
| CellTiter-Glo® 3D Cell Viability Assay | Promega | Cat# G9682 |
| 2,7-Dichlorodihydrofluorescein diacetate | Cayman Chemical | Cat# 85155 |
| Water for LC-MS | Honeywell Burdick & Jackson | Cat# 600-30-78 |
| Acetonitrile for LC-MS | Honeywell Riedel-de Haën™ | Cat# 60-046-515 |
| Methanol for LC-MS | Honeywell Burdick & Jackson | Cat#: 60-009-61 |
| Metabolomics Quality Control (QC) kit | Cambridge Isotope Labs | Cat# MSK-QC-KIT |
| Atlantis Premier BEH Z-HILIC VanGuard FIT column | Waters | SKU: 186009990 |
| Polybrene | Sigma | Cat# TR-1003-G |
| 3rd Generation Packaging System Mix | Applied Biological Materials | Cat# LV053 |
| Complete protease inhibitor cocktail | Roche | Cat# 11873580001 |
| Phosphatase inhibitor cocktail 2 | Sigma | Cat# P5726 |
| Coomassie Plus (Bradford) protein assay | Thermo Fisher | Cat# 23236 |
| Crystal Violet Solution | Sigma | Cat# HT90132 |
| PlasmoTest™ Mycoplasma Detection Kit | InvivoGen | Cat# rep-pt1 |
| **Software** |  |  |
| GraphPad Prism 10.0 | https://www.graphpad.com/ | GraphPad |
| Odyssey Fc Imaging system (LI-COR) - LI-COR Image Studio software v6.0 | https://www.licor.com/bio/image-studio/ | LI-COR |
| M5 Spectramax plate reader –  SoftMaxPro Software 6.2.1 | https://www.moleculardevices.com/  products/microplate-readers/acquisitionand-  analysissoftware/softmax-pro-software | Molecular Devices |
| MetaboAnalyst | https://www.metaboanalyst.ca/ | Open-source online tool |
| MZmine software 3.53 | https://mzio.io/ | Open-source software |
