# Supplementary material for: Targeting GSTZ1 Sensitizes KRASG12C-Mutant Lung Cancer Cells by Overcoming Glutathione and Glycolysis Pathway Rewiring
Source: Cancer Res Commun. 2026 Jun 11;6(6):1376–87. doi: 10.1158/2767-9764.CRC-25-0698 (PMC13254912; doi:10.1158/2767-9764.CRC-25-0698)
Supplement: Figure S1 — shows the effect of GSTZ1 silencing or knockout on cell viability and sensitivity to KRAS G12C inhibitors across KRAS G12C NSCLC lines in 2D and 3D, and their corresponding immunoblots. It also includes DepMap correlation and survival analysis. [file crc-25-0698_figure_s1_suppsf1.docx]

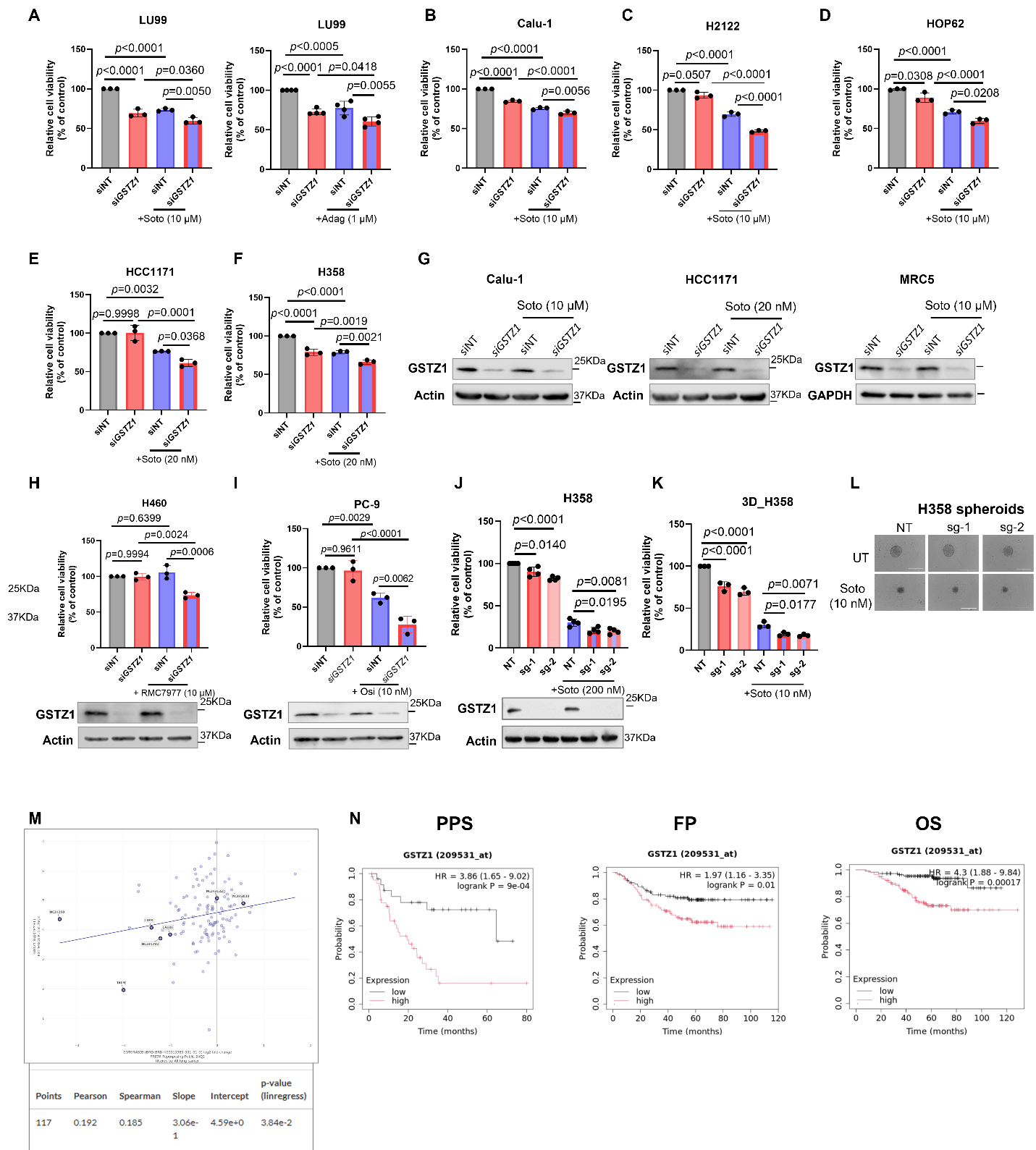


**Figure S1.** ***GSTZ1* knockdown sensitizes a panel of *KRAS^G12C^*-mutant NSCLC cell lines to KRAS^G12C^ inhibitors.** (**A-F**) Relative cell viability of LU99 (**A**), Calu-1 (**B**), H2122 (**C**), HOP62 (**D**), HCC1171 (**E**), and H358 (**F**) transfected with non-targeting (NT) or si*GSTZ1* for 24 hours and treated with sotorasib (Soto, *N* = 3) or adagrasib (Adag, *N* = 4) at indicated doses for additional 72 hours in 96-well microtiter plates. Cell viability was measured using CellTiter-Glo luminescent cell viability assay. (**G**) Immunoblotting of GSTZ1 protein levels in Calu-1, HCC1171, and MRC5 cells following *GSTZ1* knockdown and sotorasib treatment. *N* = 3. Relative cell viability of H460 (**H**) and PC-9 cells (**I**) along with the immunoblotting of GSTZ1 protein levels. *N* = 3. (**J**) Cell viability of H358 cells treated with Soto (200 nM) upon *GSTZ1* knockout for 72 hours. *GSTZ1* knockout was also confirmed in H358 using two individual sgRNAs, sg-1 and sg-2, and non-targeting sgRNA control (NT). *N* = 4. *GSTZ1* knockdown validated by immunoblotting. (**K**) Cell viability of H358 cells with NT and *GSTZ1* knockout in 3D culture enabled using 96-well spheroid microplates. Soto (10 nM) was incubated with spheroids for 72 hours after 48-hour spheroid formation. Cell viability was measured using 3D CellTiter-Glo luminescent cell viability assay. *N* = 3. (**L**) Representative brightfield images of 3D H358 spheroids shown at scale bars of 400 µm. (**M**) DepMap-based correlation analysis between *GSTZ1* gene dependency and sotorasib activity across lung cancer cell lines. (**N**) Kaplan-Meier analysis of *GSTZ1* gene expression and overall survival (OS), post‑progress survival (PPS), and first progression (FP). Data are mean ± SD and one-way ANOVA and Tukey’s post hoc test was used for statistical analysis.
