# Supplementary material for: Targeting GSTZ1 Sensitizes KRASG12C-Mutant Lung Cancer Cells by Overcoming Glutathione and Glycolysis Pathway Rewiring
Source: Cancer Res Commun. 2026 Jun 11;6(6):1376–87. doi: 10.1158/2767-9764.CRC-25-0698 (PMC13254912; doi:10.1158/2767-9764.CRC-25-0698)
Supplement: Figure S2 — shows the metabolomics workflow and the effect of GSTZ1 knockdown with or without sotorasib on glutathione, glycolytic, and lipid pathways with enriched pathway annotations. DepMap-based gene expression analysis shows correlation of GSH and GSH metabolism with GSTZ1-targeting effect. [file crc-25-0698_figure_s2_suppsf2.docx]

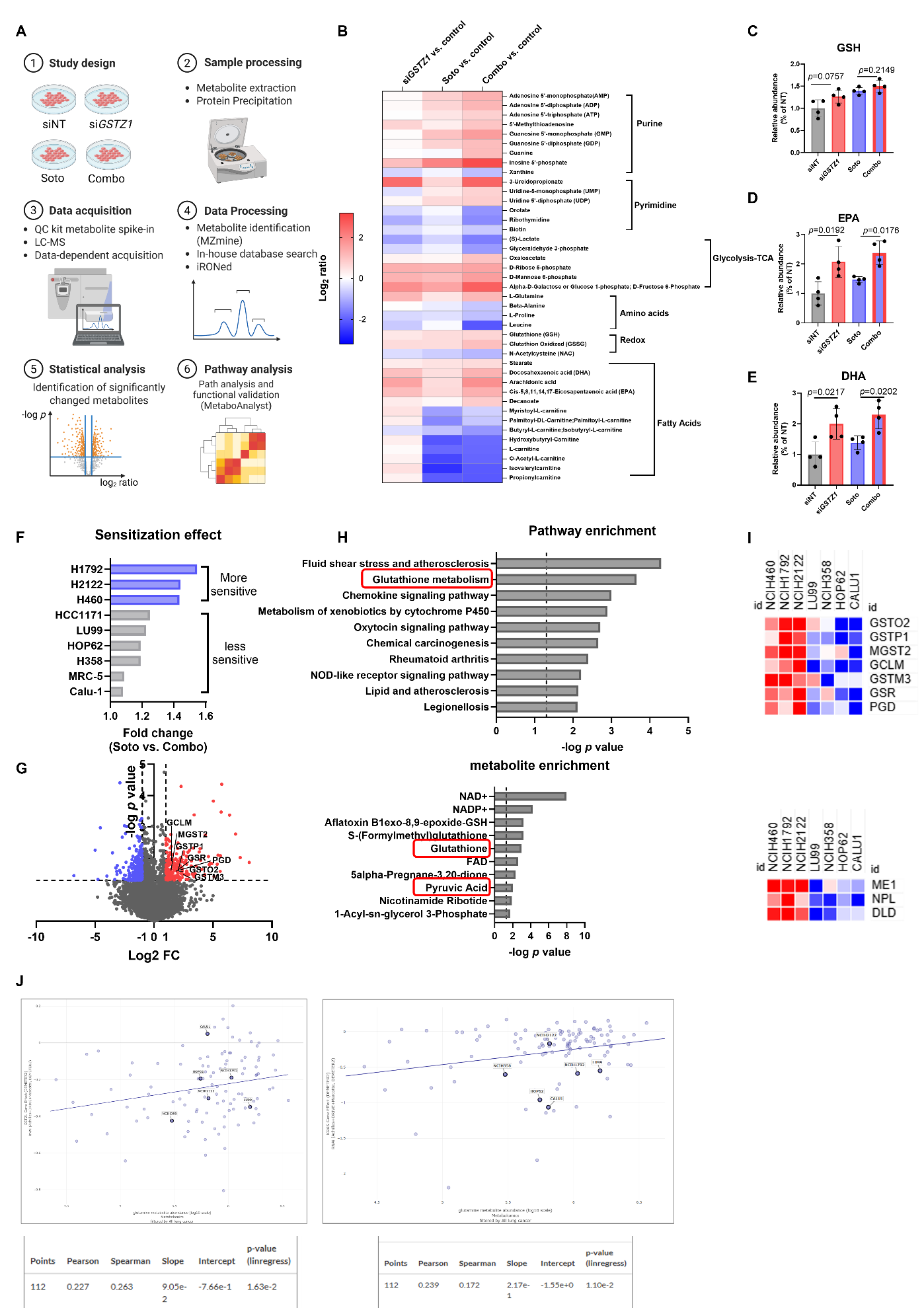


**Figure S2. *GSTZ1* knockdown alters metabolic pathways in *KRAS^G12C^*-mutant NSCLC cells.** (**A**) Schematic overview of metabolomics workflow. (**B**) Heatmap showing log2 ratio of annotated metabolites in H1792 cells treated with si*GSTZ1*, Soto (1 µM), or their combination versus non-targeting control (NT). Key metabolic classes are annotated based on the significantly changed metabolites detected in the combination treatment group. (**C-E**) Quantification of glutathione (GSH) (**C**) Cis-5,8,11,14,17-Eicosapentaenoic acid (EPA) (**D**) docosahexaenoic acid (DHA) (**E**) levels. Welch’s t-test was used for comparison and data are displayed as mean ± SD. *N* = 4. (**F**) Fold change in cell viability of the sotorasib treatment group relative to the combined sotorasib and siGSTZ1 condition (Combo). (**G**) Volcano plot showing the log₂ fold change in gene expression against statistical significance calculated using Welch’s t‑test. Genes with log₂ fold change ≥ 1 or ≤ –1 and *p* < 0.05 were considered significantly altered. (**H**) Significantly altered genes were subjected to KEGG Signaling Pathway and Metabolomics Workbench Metabolites enrichment analyses using Enrichr. (**I**) Gene expression profiles associated with enriched glutathione‑metabolism pathways and the metabolites glutathione and pyruvic acid. (**J**) DepMap-based analysis of the correlation of glutamine levels with either GSTZ1 or KRAS gene dependency across lung cancer cell lines.
