# Supplementary material for: Targeting GSTZ1 Sensitizes KRASG12C-Mutant Lung Cancer Cells by Overcoming Glutathione and Glycolysis Pathway Rewiring
Source: Cancer Res Commun. 2026 Jun 11;6(6):1376–87. doi: 10.1158/2767-9764.CRC-25-0698 (PMC13254912; doi:10.1158/2767-9764.CRC-25-0698)
Supplement: Figure S3 — shows the effect of GSTZ1 silencing, along with BSO or glutor, on GSH, lactate, and ROS levels, antioxidant rescue and cell viability. [file crc-25-0698_figure_s3_suppsf3.docx]

**
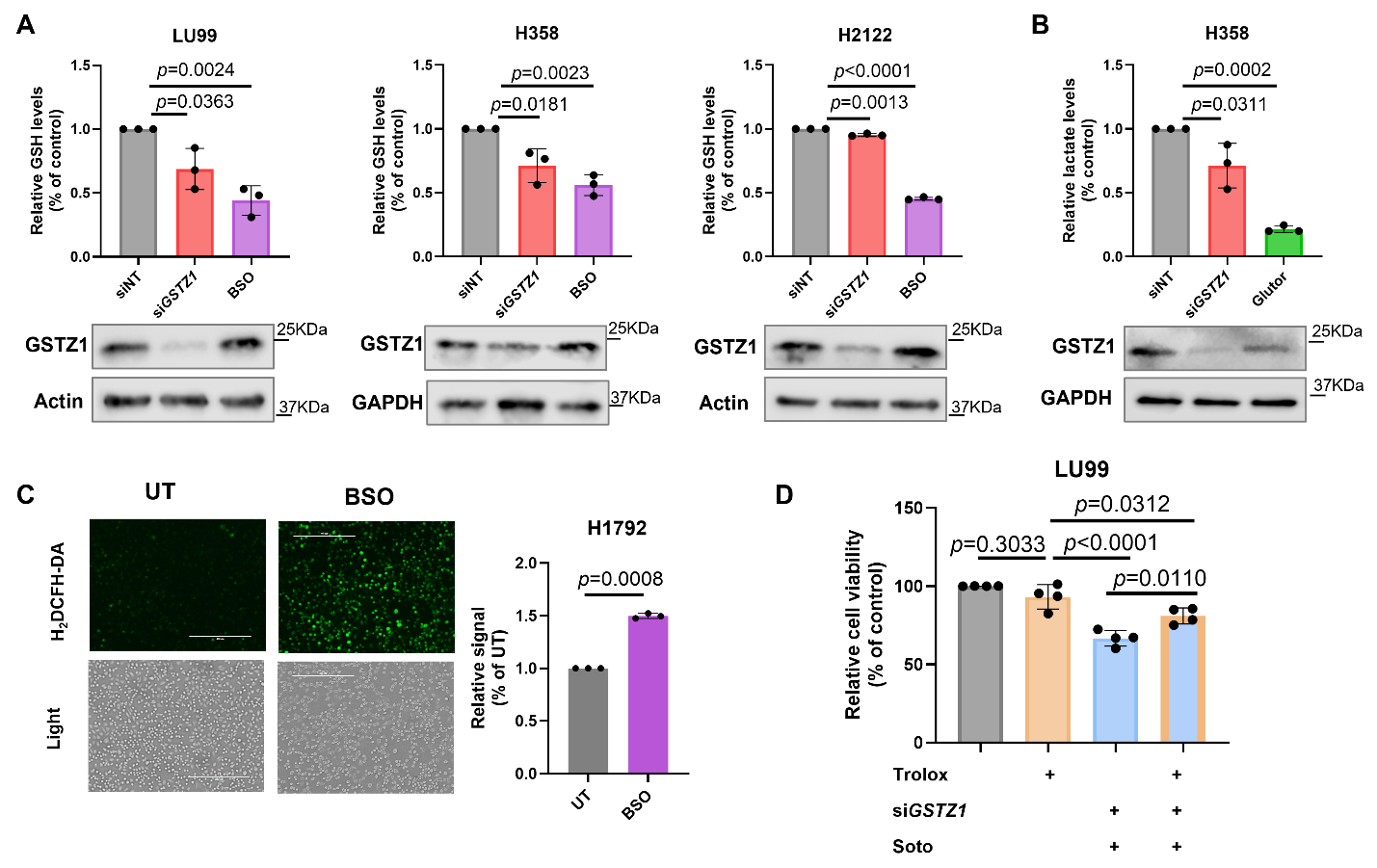
**

**Figure S3. *GSTZ1* silencing reduces GSH and lactate levels and increases ROS levels.** (**A**) GSH levels in H358 and H2122 cells treated with si*GSTZ1*, BSO (500 µM) or BSO (100 µM) in LU99 cells for 48 hours. Immunoblots confirmed *GSTZ1* knockdown. *N* = 3. (**B)** Lactate levels in H358 cells following si*GSTZ1* or glutor (50 nM) treatment for 48 hours. *N* = 3. (**C**) ROS levels visualized by H₂DCFH-DA staining in H1792 cells after BSO (500 µM) treatment and H₂DCFH-DA fluorescence intensity was measured using a microplate reader at the excitation 485 nm and emission 530 nm. *N* = 3. (**D**) Viability of LU99 cells treated with si*GSTZ1* and/or Soto (1 µM), with or without antioxidant Trolox (50 µM). *N* = 4. Data represent mean ± SD and analyzed by one-way ANOVA.
