# Supplementary material for: Targeting GSTZ1 Sensitizes KRASG12C-Mutant Lung Cancer Cells by Overcoming Glutathione and Glycolysis Pathway Rewiring
Source: Cancer Res Commun. 2026 Jun 11;6(6):1376–87. doi: 10.1158/2767-9764.CRC-25-0698 (PMC13254912; doi:10.1158/2767-9764.CRC-25-0698)
Supplement: Figure S4 — shows the effect of pharmacologic inhibition of glutathione synthesis with BSO or glycolysis with glutor on KRAS G12C inhibitor cytotoxicity in LU99 and H2122 cells. [file crc-25-0698_figure_s4_suppsf4.docx]

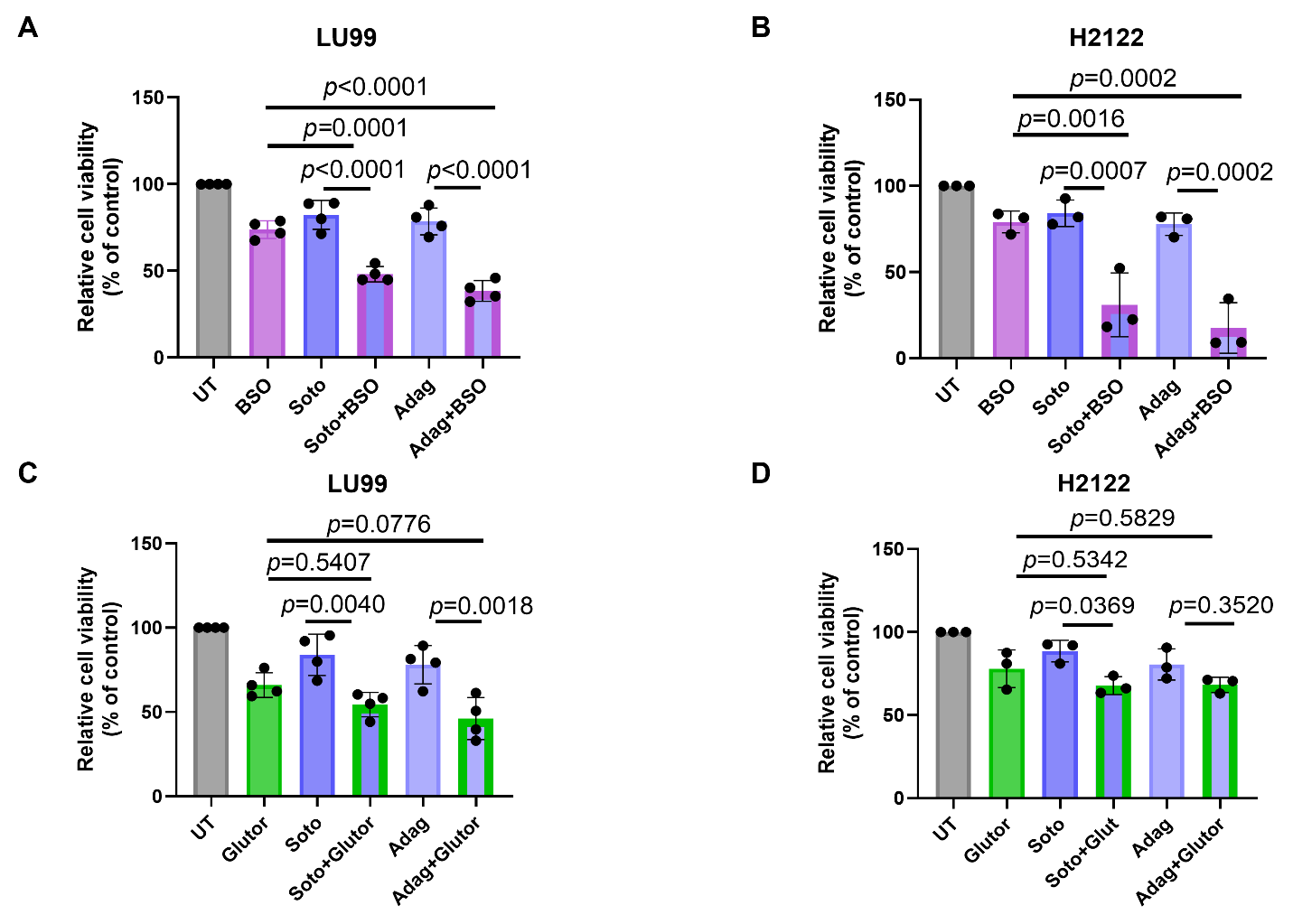


**Figure S4. Targeting glutathione and glycolysis pathways enhances KRAS inhibitor efficacy.** (**A, C**) Cell viability of LU99 following treatment with BSO (100 µM) or glutor (5 nM) alone or in combination with Soto (10 µM) or Adag (1 µM). *N* = 4. (**B, D**) Cell viability of H2122 cells following treatment with BSO (500 µM) or glutor (50 nM) alone or in combination with Soto (10 µM) or Adag (1 µM). *N* = 3. Data were analyzed by one-way ANOVA and bars represent mean ± SD.
