# Supplementary material for: Targeting GSTZ1 Sensitizes KRASG12C-Mutant Lung Cancer Cells by Overcoming Glutathione and Glycolysis Pathway Rewiring
Source: Cancer Res Commun. 2026 Jun 11;6(6):1376–87. doi: 10.1158/2767-9764.CRC-25-0698 (PMC13254912; doi:10.1158/2767-9764.CRC-25-0698)
Supplement: Figure S5 — shows the effect of GSTZ1 loss or combined metabolic inhibition on p-AMPK, p-AKT and p-ERK signaling across cell lines. [file crc-25-0698_figure_s5_suppsf5.docx]

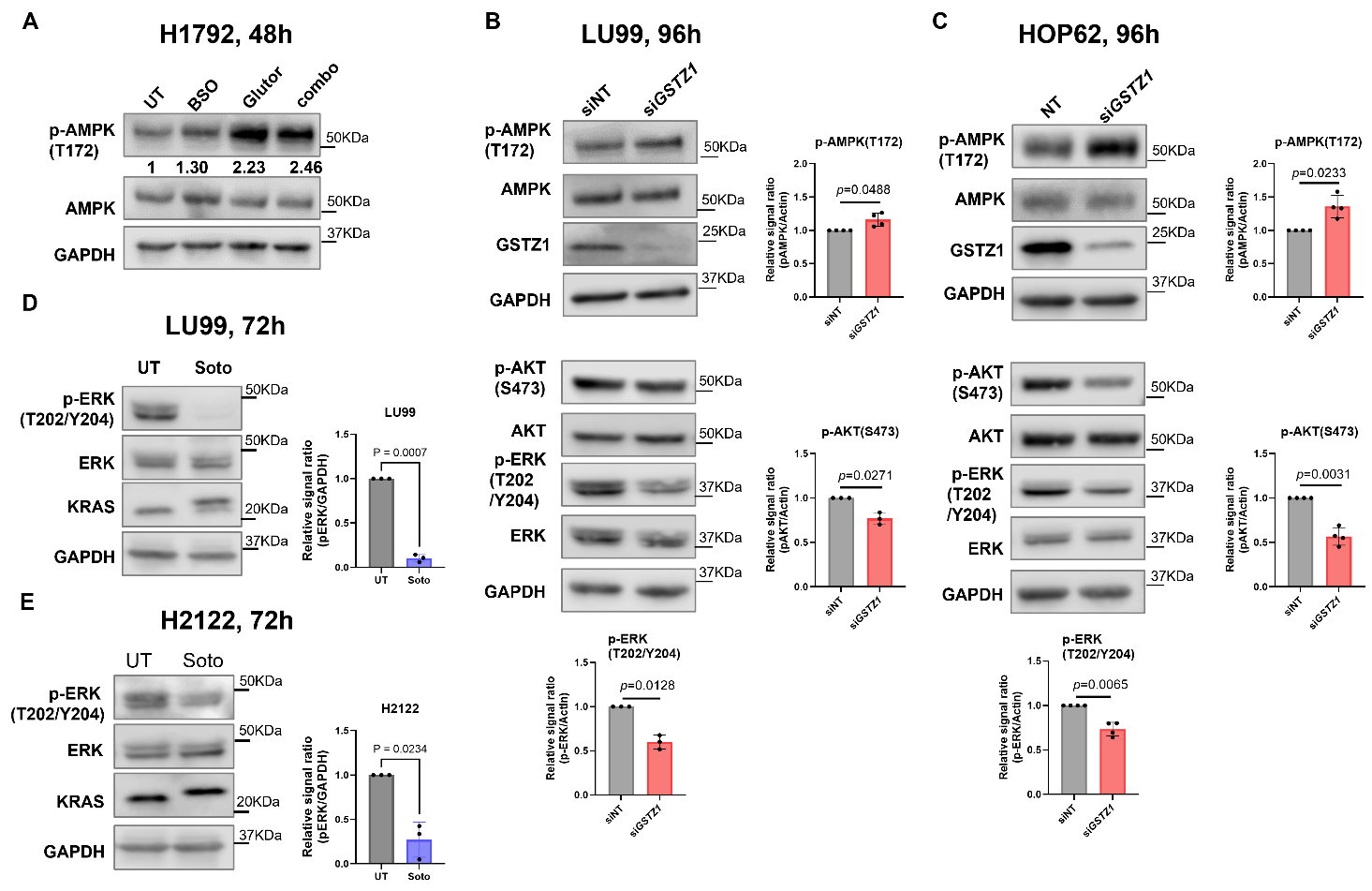


**Figure S5. Targeting glutathione and glycolysis pathways recapitulates the effect of *GSTZ1* knockdown on AMPK phosphorylation and survival signaling.**

(**A)** Immunoblot of AMPK activation in H1792 cells after treatment with BSO (500 µM), glutor (50 nM), or combination, as evidenced by the increased phosphorylation of AMPK at threonine 172 (p-AMPK T172). Quantification and fold change relative to DMSO treatment (UT) was shown below. (**B**) Immunoblots and quantification in LU99 and HOP62 cells treated with si*GSTZ1* for 96 hours. Quantification of p-AMPK (T172, *N* = 4), p-AKT (S437, *N* = 3), and p-ERK (T202/Y204, *N* = 3). (**C**) Immunoblots and quantification in HOP62 cells treated with si*GSTZ1* for 96 hours. Quantification of p-AMPK (T172, *N* = 4), p-AKT (S473, *N* = 4), and p-ERK (T202/Y204, *N* = 4). Quantification of phospho-protein signals was done relative to actin. (**D**) Effect of Soto (10 µM) on p-ERK (T202/Y204), ERK, and KRAS in LU99. *N* = 3. (**E**) Effect of Soto (10 µM) on p-ERK (T202/Y204), ERK, and KRAS in H2122. *N* = 3. Statistical analysis was conducted by using Welch’s t-test. Data were shown as mean ± SD.
